# Gaps and uncertainties in the management of acute pancreatitis: a scoping review and quality assessment of clinical practice guidelines

**DOI:** 10.1016/j.eclinm.2025.103216

**Published:** 2025-05-15

**Authors:** Sivesh K. Kamarajah, Vignesh Gopalan, Zarnigar Khan, Daniel M. Baker, Amy Lucas, David Hawkins, Stacey Munnelly, Marianne Hollyman, Laura Magill, Matthew J. Lee

**Affiliations:** a Department of Applied Health Sciences, School of Health Sciences, College of Medicine and Health, University of Birmingham, Birmingham, United Kingdom; b NIHR Global Health Research Unit on Global Surgery, University of Birmingham, Birmingham, United Kingdom; c Somerset NHS Foundation Trust, Musgrove Park Hospital, Taunton, United Kingdom; d Department of Surgery, Sheffield Teaching Hospitals NHS Foundation Trust, Sheffield, United Kingdom; e Department of Surgery, Leeds Teaching Hospitals NHS Foundation Trust, Leeds, United Kingdom; f GUTS UK Charity, London, England; g Department of Gastroenterology, Manchester Royal Infirmary, Manchester University NHS Foundation Trust, Manchester, United Kingdom

**Keywords:** Pancreatitis, Universal health coverage, Rehabilitation, Outcomes

## Abstract

**Background:**

Universal health coverage (UHC) emphasises equitable care for all, without financial hardships and healthcare professionals use clinical practice guidelines (CPGs) to inform service delivery. With rising burden of acute pancreatitis worldwide, the recent James Lind Alliance highlighted knowledge gaps exist within current pathways. This scoping review and quality assessment aims to determine gaps and uncertainties in CPGs for the management of acute pancreatitis.

**Methods:**

We conducted a scoping review, through a Arksey and O'Malley five-staged process. Electronic databases (MEDLINE, Embase, CINAHL, PsycINFO), targeted websites, and reference lists were used to identify eligible CPGs on acute pancreatitis on February 22, 2024, and updated on March 27, 2025. Drawing from the UHC effective coverage framework, we mapped twelve indicators to a matrix representing health service types (i.e., promotion, prevention, treatment, rehabilitation). Corresponding strength and quality of each recommendation were extracted and quality of overall CPGs was assessed using AGREE-II.

**Findings:**

Of 22 CPGs identified, 11 were published over the past decade and 17 (77.3%) were from high income countries only. Only two guidelines included experts in rehabilitation and policymakers. 718 recommendations were made and reported across: (i) prevention (n = 3; 2 studies); (ii) treatment (n = 696; 22 studies); and (iii) rehabilitation (n = 19; 4 studies). There were no recommendations made around promotion or palliation. Of the twelve indicators, only obesity prevention or weight management were not covered in any of the guidelines. Within the treatment domain, majority of recommendations focussed on early in-hospital management (n = 215, 30.9%). Of these 337 (48.4%) were graded as strong, but only 125 were (17.9%) were supported by high-level evidence. There were 19 recommendations on rehabilitation across two indicators, which were follow-up care (n = 17) and diagnosis and management of new long-term conditions (n = 2). Of these recommendations, 325 (49.7%) were strong, 27 (4.1%) were moderate, and 171 (26.1%) were weak recommendations, respectively. However, only 109 (16.7%) recommendations were supported by high-level evidence and the majority (n = 230, 35.2%) had moderate-level evidence.

**Interpretation:**

Recommendations on care of AP focus on the in-hospital management, with limited supporting recovery or rehabilitation after acute pancreatitis. Although quality of guidelines has improved over time, discrepancies exist between strength of recommendations and quality of evidence across these domains. Inequity around clinical guidance for promotion, prevention and rehabilitation in patients, serves as a call to action for research to improve long-term outcomes for these patients, aligned to global priorities.

**Funding:**

SK was funded by the NIHR Doctoral Research Fellowship (NIHR303288). The views expressed in this publication are those of the author(s) and not necessarily those of the NIHR or the UK government. The funders had no role in study design, data collection, data analysis, data interpretation, writing of the report, or the decision to submit the paper for publication.

**Research in context**

**Evidence before this study**

Acute pancreatitis (AP) is a common emergency presentation around the world and is associated with a significant cause of morbidity and healthcare burden worldwide, yet its management continues to be inconsistent due to variations in clinical practice guidelines (CPGs). While several guidelines have been developed to standardise treatment approaches, there is considerable disparity in recommendations across different regions and healthcare settings. Previous research has highlighted deficiencies in adherence to guidelines and their implications for patient outcomes, but no comprehensive review has systematically examined gaps across all available guidelines. The James Lind Alliance Priority Setting Partnership identified key uncertainties in AP management, underscoring the need for further research into best practice, but how these knowledge gaps are reflected in guideline recommendations has not been formally evaluated.

**Added value of this study**

This study provides a comprehensive scoping review assessing global CPGs for AP, identifying critical gaps in the strength and quality of recommendations. The findings indicate that existing guidelines overwhelmingly focus on in-hospital treatment, while areas such as rehabilitation and long-term follow-up remain underrepresented. In particular, the study highlights the misalignment between recommendation strength and the quality of underlying evidence, with nearly half of strong recommendations based on low- or moderate-quality evidence. Furthermore, most guidelines originate from high-income countries (77.3%), raising concerns regarding their applicability to low-resource settings. By mapping recommendations against the Universal Health Coverage (UHC) effective coverage framework, the study illustrates the fragmented nature of guideline recommendations, with limited emphasis on prevention, recovery, and long-term care.

**Implications of all the available evidence**

This review and quality assessment demonstrates the pressing need for greater standardisation and harmonisation of AP guidelines, ensuring that recommendations are underpinned by robust evidence and applicable across diverse healthcare systems. The findings reinforce the importance of multidisciplinary involvement in guideline development, particularly incorporating rehabilitation specialists, policymakers, and patients with lived experience. Future research should prioritise high-quality trials addressing controversial areas, such as early fluid resuscitation strategies, antibiotic use, and structured rehabilitation interventions. Additionally, greater attention should be paid to long-term patient outcomes, including the prevention of recurrent pancreatitis and the management of complications such as pancreatic exocrine and endocrine insufficiency.

## Introduction

Acute pancreatitis (AP) is a gastrointestinal condition that affects up to three million patients each year around the world.^1^, ^2^, ^3^ The incidence of AP has increased over the past decade and is one of the most common emergency presentations requiring hospital admission around the world.^4^, ^5^, ^6^ Approximately 10% of patients present with severe AP and up to one-in-ten patients die during their hospital admission.^7^ Common causes for acute pancreatitis include gallstones and excess alcohol intake. These risk factors are increasingly common in patients from poor socioeconomic status^2^ and the overall burden remains high with the aging population.^2^ In the UK, acute pancreatitis impacts 40,000 patients and annual costs to the healthcare system in managing these patients are as high as £200 million.^8^^,^^9^ AP is a relatively neglected area of clinical research^10^ and the evidence deficit was highlighted by the James Lind Alliance Priority Setting Partnership.^9^

Clinical practice guidelines (CPGs) are tools used to translate research evidence into clinical practice.^11^ They are defined as recommendations based on a rigorous review of evidence and an evaluation of alternative care options.^11^^,^^12^ It is considered good practice to demonstrate adherence to clinical guidelines and evidence-based recommendations, which are associated to improved outcomes in patients.^11^ At present, CPGs are often developed with a focus on treatment and cost-effectiveness, without regard for a whole systems approach to maximise universal health coverage.^13^ Therefore, clinical recommendation may not always be applicable or beneficial to patients in their entire clinical pathway, extending to rehabilitation or recovery.^9^^,^^14^ There is growing emphasis on measuring how care is provided across the spectrum of service utilisation.^15^^,^^16^ CPGs therefore need to provide comprehensive recommendations on disease management across this spectrum, from prevention to palliation, to ensure high-quality care.^17^^,^^18^ The management of AP is intricately linked to its underlying pathophysiological mechanisms, including pancreatic inflammation, necrosis, and systemic inflammatory response syndrome (SIRS). These processes influence decisions regarding early fluid resuscitation, nutritional support, and antibiotic administration. Understanding how current guidelines address–or fail to address–these fundamental aspects of disease progression is crucial to optimising treatment strategies. Despite presence of multiple CPGs on AP, there remains a knowledge gap around understanding best practices to implement. This inherently leads to variation in clinical practice globally,^7^^,^^19^^,^^20^ affecting patient outcomes, and resource use across health systems.

To bridge this knowledge gap, we performed a scoping review and quality assessment of CPGs of adult patients with acute pancreatitis. The objectives of this scoping review were: (i) to develop a conceptual framework of key pathway domains; ii) to assess representation of pathway domains in CPGs; (iii) to assess the strength of recommendations and quality of evidence related to domains; and (iv) quality of CPGs and process measures in development. Understanding differences between different CPGs will help generate new areas of future research efforts and policymaking nationally and regionally towards standardised management of patients with AP.

## Methods

This scoping review was conducted according to the Arksey and O'Malley (2005)^21^ five-staged process and the PRISMA Extension for Systematic Reviews Checklist (Appendix p 3–4, Table S1).^22^ The overall process of this systematic review is summarised in Figure S1.

### Stage 1: research question

What are the current gaps and uncertainties of clinical practice guidelines for the management of adult patients with acute pancreatitis, both in-hospital and follow-up?

### Stage 2: literature search

We developed a systematic search strategy to identify relevant literature. Systematic searches were performed in MEDLINE (Ovid), Embase (Ovid), PsycINFO (Ovid), CINAHL Plus (EBSCO), and Cochrane Library to identify relevant studies between 1st January 2000 and March 1, 2025. The initial search was done on February 22, 2024, and updated on March 27, 2025. This was done to restrict to CPGs in the 21st century owing to changes in healthcare systems over time The full search strategy, developed with an information specialist, is shown in the Appendix (Appendix p 5, Table S2). To supplement the database search, we hand-searched reference lists of any clinical practice guidelines that we found, to maximise capture of relevant guidelines.

### Stage 3: literature selection

All studies identified in the scoping review were extracted into Microsoft Excel. The study screening was delivered over a two-staged process by two independent reviewers (SKK, VG). In stage 1, all the titles and abstracts were screened by two authors (SKK, VG). Disagreements were resolved by consensus, and in the case that consensus could not be reached, the senior author (MJL) arbitrated. Once agreement was reached on the included studies, the titles and abstracts were screened again by two more authors, prior to retrieving full texts. Inclusion criteria were: (i) clinical practice guideline for the management of adult patients (aged ≥18 years) with acute pancreatitis; (ii) provide guidance into more than one facet of investigations, diagnosis and management (i.e., guidance on nutritional support and antibiotic prescribing). Where more than one CPG originate from a group, the most up to date CPG was included. The exclusion criteria were: (i) non-English papers; (ii) secondary review of clinical guidelines; (iii) clinical practice guidelines focussing on chronic pancreatitis and severe acute pancreatitis only; and (iv) not most recent guideline or position statement by date of publication (supplemented by newest version by the same government or organisation). Where multiple guidelines were identified from the same group, country, or authorship, the most recent guideline was included to avoid duplication and potential bias due to overlapping recommendations. Specifically, guidelines from the same lead author or group published sequentially were carefully screened to ensure only the most updated, authoritative version was included in our analysis. This approach reduced redundancy, minimised bias due to overlapping authorship, and ensured that our analysis accurately reflects the latest guidance provided by authoritative expert groups.

### Stage 4: data extraction

A pre-designed data collection form was developed using Microsoft Excel to extract data on baseline characteristics of CPGs (Appendix p 6, Table S3). Details of the guideline development process were collected, which include: (i) number of countries involved in the development; (ii) specialty or expertise of members involved; and (iii) process of guideline development (i.e., literature review, consensus process through Delphi).

To measure coverage of CPGs along the patient pathway in patients with acute pancreatitis, we adapted the UHC effective coverage framework^23^ which have been previously published and validated.^13^ Briefly, the UHC framework was developed in 2014 by the World Health Organisation and World Bank This framework emphasises the importance of providing services for individuals' health needs throughout their lifespans and quantifying effective coverage of interventions delivered by health systems. Conceptually, effective coverage unites intervention need, use, and quality into a single metric, representing the proportion of health gain that could be potentially received from an intervention relative to what is actually experienced.^24^^,^^25^ At the health-system level, effective coverage aims to capture the fraction of total potential health gains actually delivered relative to what a health system could have theoretically delivered. Broadly, this framework includes five broad domains on health service types such as: (i) promotion; (ii) prevention; (iii) treatment; (iv) rehabilitation; and (v) palliation. Within each of these UHC domains, we identified and prioritised a set of indicators to measure coverage of the CPGs for acute pancreatitis. This process was developed through several rounds of consultations with the Study Management Group, which include a diverse group of clinicians, health service researchers and methodologist with expertise in pancreatitis research. Indicators used to measure promotion and prevention were availability of recommendations on smoking, alcohol and obesity since these are well-recognised risk-factors for acute pancreatitis.^1^^,^^26^ In the context of acute pancreatitis, promotion was defined as indicators aimed at improving overall health. However, prevention was defined as indicators aimed at reducing further recurrence of disease. We recognise some indicators may overlap in the context of promotion and prevention such as smoking and alcohol cessation.

For the treatment domain, seven indicators were identified: (i) diagnosis of disease; (ii) assessment of severity; (iii) early in-hospital management; (iv) late in-hospital management; (v) interventional management; (vi) intensive care unit management; and (vii) organisation or model of care. For the rehabilitation domain, key indicators were: (i) follow-up care; and (ii) diagnosis and management of new long-term conditions such as exocrine insufficiency. Matrix of indicators and domains and their definition are described in Appendix (p 7–8; Tables S4 & S5). Strength of recommendations were defined as strong, moderate, weak, very weak or none, or not reported according to the Grading of Recommendations, Assessment, Development, and Evaluations (GRADE) framework.^27^ Grading the quality of evidence underpinning each were defined as high, moderate, low, or very low. In the instance where no grading of the quality was performed for each recommendation within a CPG, this was extracted as ‘not reported’ in the analysis.

### Stage 5: data synthesis

For each CPG, each recommendation was extracted by two reviewers (SKK, VG). The recommendation was mapped to a domain, and corresponding strength and quality of evidence were recorded. Each recommendation was grouped in preparation for narrative synthesis in a three-staged process. First, two reviewers (SKK, VG) coded and summarised each component of the recommendations against the developed conceptual framework. Both reviewers (SKK, VG) then reviewed codes, with any disagreements resolved through discussion with a third reviewer (MJL) and Study Management Group as required. Secondly, we generated sub-domains of features for each key domain, to structure coded recommendations. Finally, we grouped the sub-domains based on these domains, using a framework developed through discussion between the Study Management Group, as described above. For each recommendation, the association between the strength and quality of evidence were also reported.

### Quality assessment of CPGs

The quality of the CPGs was evaluated using the Appraisal of Guidelines for Research and Evaluation (AGREE-II).^28^ The AGREE-II instrument consists of 23 questions in six domains. It uses a Likert scale, ranging from one to seven for each question, and a final score is given as the percentage of the maximum possible score. This process was performed by four independent members of the study team (SKK, VG, DMB, ZK), to robustly understand quality of the CPGs. To derive the final AGREE-II scores, the average was calculated across four reviewers in each study. An analysis was performed to report the inter-evaluator agreement AGREE-II scores across clinical practice guidelines. To do so, we calculated the Intraclass Correlation Coefficient (ICC) using a two-way random-effects model for absolute agreement.^29^ Given that each guideline was independently rated by four evaluators, we employed ICC (2, 1) to evaluate single-rater reliability and ICC (2, k) to assess the reliability of the average rating across evaluators. The ICC was computed using the psych package in R. Interpretation of the ICC values followed established thresholds: <0.40 (poor agreement), 0.40–0.59 (fair agreement), 0.60–0.74 (good agreement), and ≥0.75 (excellent agreement). A high ICC (2, k) would indicate strong consistency among evaluators, supporting the robustness of our scoring approach. In contrast, a lower ICC would suggest variability in evaluator interpretation, necessitating further standardisation of rating criteria.

### Statistical analysis

To evaluate trends in guideline quality over time, linear regression analysis was performed using guideline publication year as the independent variable and AGREE-II scores as the dependent variable. The strength and significance of this relationship were assessed using the coefficient of determination (r^2^) and p-values, with statistical significance set at p < 0.05. This analysis was conducted using the R statistical software (version 3.2.2), to systematically quantify whether newer guidelines demonstrated improved methodological quality.

### Patient and public involvement

This scoping review and quality assessment had input from a patient and public involvement (PPI) group who led the James Lind Alliance Priority Setting Partnership (AL, DH). The PPI group included eight patients with lived experience of acute pancreatitis who have received care in hospitals to ensure the key conclusions drawn were meaningful and relevant. This PPI group, with a named patient lead (AL, DH) were involved in the design of this scoping review protocol and interpretation of study findings. All PPI activity and recommendations were reported according to the GRIPP-2 guidance.^30^

### Ethics

Ethics approval was not needed to conduct this scoping review because it is based on existing literature in the public domain.

### Role of the funding source

This study received no formal funding. SK was funded by the NIHR Doctoral Research Fellowship (NIHR303288). The funders had no role in study design, data collection, data analysis, data interpretation, writing of the report, or the decision to submit the paper for publication. All authors had access to the data and were responsible for the decision to submit this scoping review for publication.

## Results

### Literature search

**Fig. 1:**
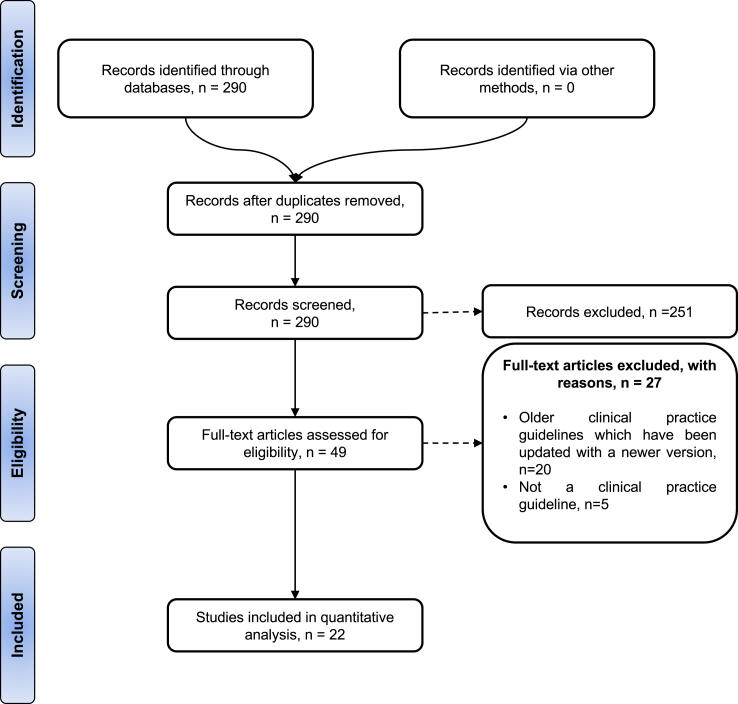
PRISMA flowchart summarising the systematic review.

The scoping review included 22 CPGs that met the inclusion criteria, of which three originated from overlapping authors or expert groups. However, on careful content analysis, all these guidelines were distinct, avoiding redundancy and preserving methodological rigour. Fig. 1 presents the PRISMA flow chart documenting the study selection process and Table 1 presents the characteristics of included CPGs.

**Table 1:** Summary of the included studies in the scoping review.

Characteristics of all clinical practice guidelines (n = 22)	N (%)
**Year of publication**	
2001–2005	2
2006–2010	5
2011–2015	4
2016–2020	7
2021–2024	4
**Country**	
Canada	1
China	1
Germany	1
Italy	3
Japan	2
Poland	1
South Korea	1
Spain	1
Taiwan	1
United Kingdom	1
United States of America	2
International partnerships	
Europe only	1
North America only (i.e., Canada, United States)	1
Europe and North America	1
Multi region collaboration	1
Not reported	3
**Universal health coverage domains**	
Promotion	0
Prevention	2
Treatment	22
Rehabilitation	4
Palliation	0
**Expertise involved in guideline development**	
Surgery	12
Gastroenterology	17
Pancreatology	8
Emergency care	3
Critical or intensive care	4
Anaesthetic care	3
Nutrition or dietetics	3
Radiology (imaging)	6
Microbiology	1
Rehabilitation or recovery	1
Policymaker	1
**Endorsement of guidelines**	
No	3
Yes	19

### Study characteristics

Of the 22 studies included, 11 were published over the past decade and 17 (77.3%) were from high income countries only. Only two studies had representation from low- and middle-income countries. Seven guidelines were developed by a group of experts in a single field and the remaining were developed by two or more expert groups. Majority of expertise were gastroenterology (n = 17), followed by surgery (n = 12) and pancreatology (n = 8). Experts in rehabilitation or recovery and policymakers were involved in one guideline each, respectively.

### Domains, strength and quality of recommendations

**Fig. 2:**
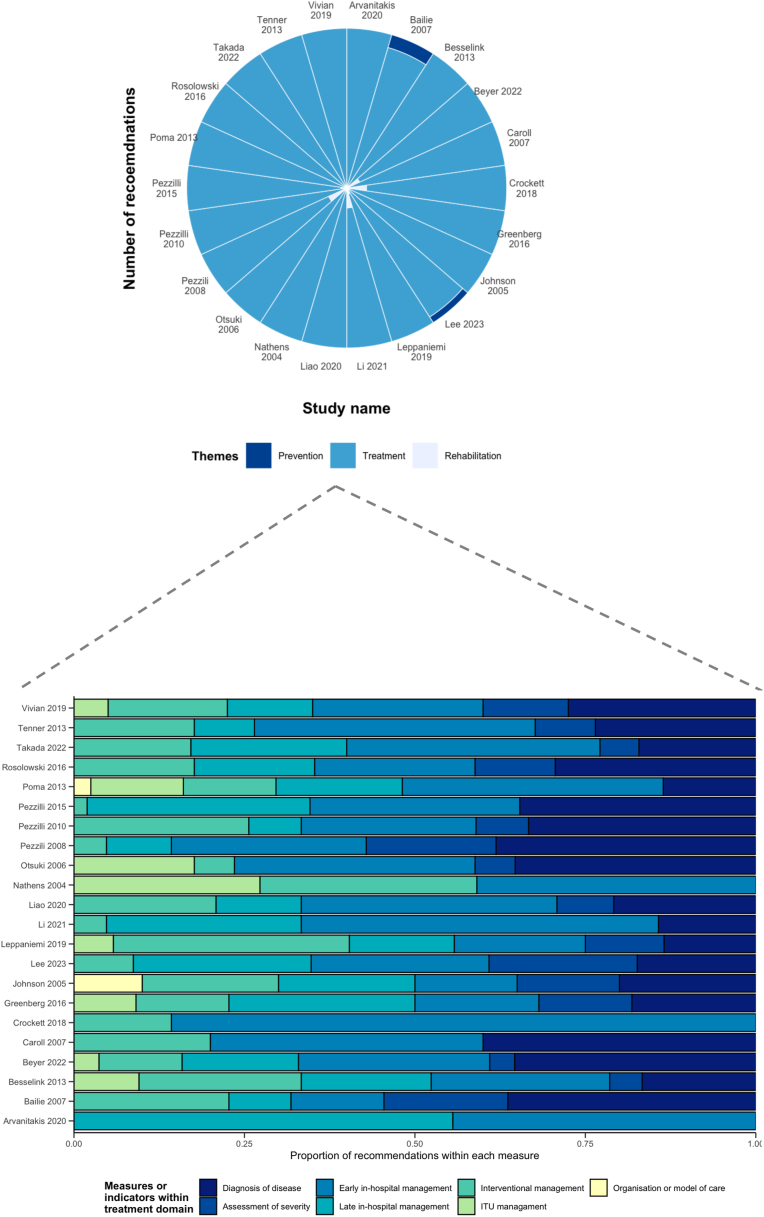
Summary of the included clinical practice guidelines and coverage of key domains along the patient pathway.

**Fig. 3:**
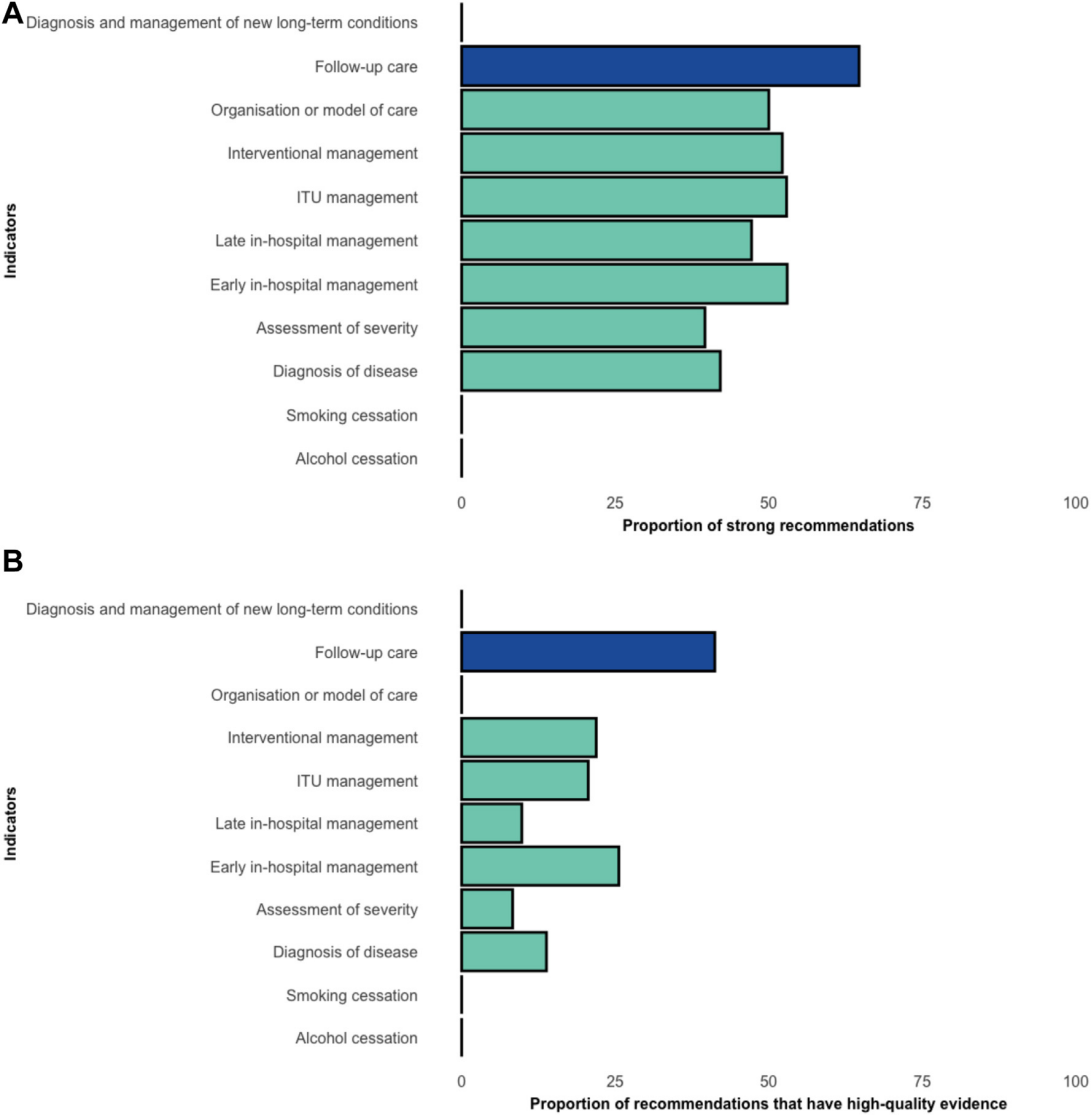
Differences in strength of recommendations and quality of evidence reported across the indicators included in the systematic review. (A) The strength of recommendation for each indicator included in the systematic review. (B) The quality of evidence supporting recommendation within each indicator.

Across the 22 CPGs, 718 recommendations were made for adult patients with acute pancreatitis. These recommendations were reported across three domains only: (i) prevention (n = 3; 2 studies); (ii) treatment (n = 696; 22 studies); and (iii) rehabilitation (n = 19; 4 studies). There were no recommendations made around promotion or palliation (Fig. 2). A summary of the recommendations by domains and indicators are presented in Table 2. Of the twelve indicators, only obesity prevention or weight management were not covered in any of the guidelines. Of these 718 recommendations, 348 (48.5%) were strong, 54 (7.4%) were moderate, and 170 (24.9%) were weak recommendations, respectively. However, only 109 (18.4%) recommendations were supported by high-level evidence and the majority (n = 256, 35.7%) had moderate-level evidence. A summary of the overall strength of recommendations and quality of evidence across the indicators are presented in Fig. 3 and by study type (Appendix p 9; Figure S1).

**Table 2:** Summary of recommendations by key domains and subdomains within clinical practice guidelines of patients with acute pancreatitis.

Key domains and subdomains	Number, n (%)
**Promotion**	**0**
**Prevention**	**3**
Alcohol cessation	2 (66.7)
Smoking cessation	1 (33.3)
Weight reduction or obesity management	0 (0)
**Treatment**	**696**
Diagnosis of disease	159 (22.8)
Overall diagnosis	111
Aetiology type	43
Imaging	5
Assessment of severity	48 (6.9)
Early in-hospital management	215 (30.9)
Fluids and analgesia	73
Nutrition	81
Antibiotics	46
Biological agents or immunotherapies	13
Other	2
Late in-hospital management	123 (17.7)
Management of complications	113
Nutrition	10
Interventional management	113 (16.2)
ERCP or EUS	48
ERCP or EUS-related complications	7
Surgery	58
Intensive care management	34 (4.9)
Organisation or model of care	4 (0.6)
**Rehabilitation**	**19**
Follow-up care	17 (89.5)
Diagnosis and management of new long-term conditions	2 (10.5)
**Palliation**	**0**

Abbreviations: ERCP: endoscopic retrograde cholangiopancreaticography, EUS:Endoscopic ultrasound.

Within the treatment domain (n = 696 recommendations), the majority of recommendations stemmed from the early in-hospital management (n = 215, 30.9%), diagnosis of disease (n = 159, 22.8%), and late in-hospital management (n = 123, 17.7%) (Appendix p 10; Table S6). Of these recommendations for the treatment domain, 337 (48.4%) were graded as strong, but only 125 were (17.9%) were supported by high-level evidence (Appendix p 11; Table S7). There were 19 recommendations on rehabilitation across two indicators, which were follow-up care (n = 17) and diagnosis and management of new long-term conditions (n = 2). Of the 19 recommendations, 11 (57.9%) were strong, but only seven (37.8%) made were supported by high-level evidence.

A sensitivity analysis was performed by the presence of gastroenterology expertise in the development in CPGs. There were no significant difference in study characteristics between guidelines with and without expertise in gastroenterology (Appendix p 12, Table S8).

### Quality of clinical practice guidelines

**Fig. 4:**
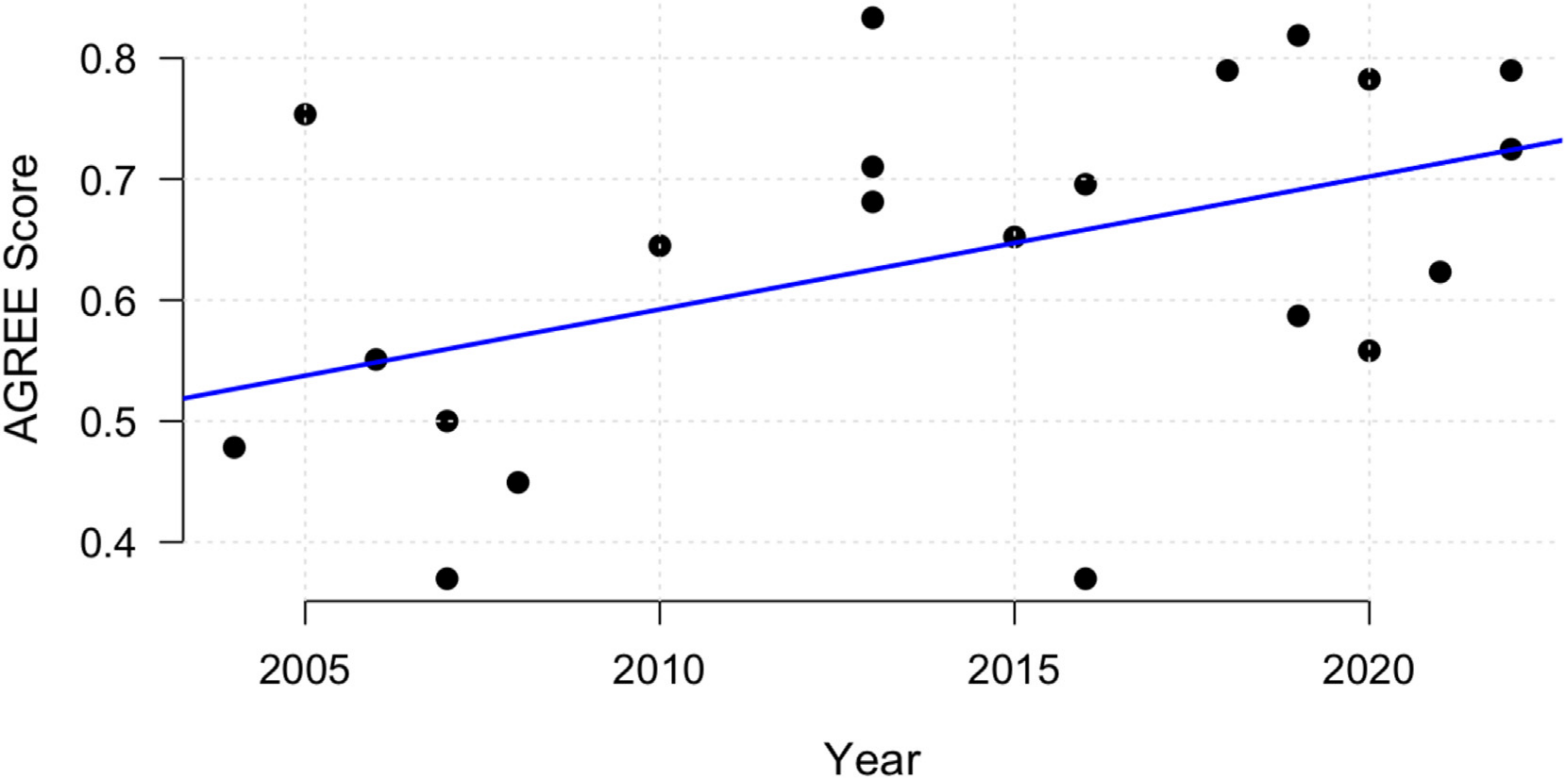
Linear regression of publication year compared to the AGREE-II score. Quality of guidelines have improved over time (p = 0.035).

A summary of the scores for each guideline according to the AGREE-II^28^ instruments are presented in Table Sx (Appendix p 13–15; Table S9). The median score is 68% (range: 57%–77%). Inter-evaluator agreement for AGREE-II scores was assessed using intraclass correlation coefficients (ICCs). The single-rater absolute agreement ICC (1,1) was 0.69 (95% CI: 0.63–0.74), indicating good but variable agreement among individual raters. The single-rater consistency ICC (2,1) was 0.70 (95% CI: 0.47–0.82), reflecting moderate reliability in independent scoring. Notably, the single-rater consistency assuming fixed raters ICC (3,1)] was higher at 0.81 (95% CI: 0.77–0.85), suggesting greater stability when the same evaluators are used across guidelines. When considering the average rating across the four evaluators, agreement improved substantially. The average-rater absolute agreement ICC (1,k) was 0.90 (95% CI: 0.87–0.92), indicating excellent reliability of aggregated scores. Similarly, the average-rater consistency ICC(2,k) was 0.90 (95% CI: 0.78–0.95), demonstrating strong agreement across evaluators. The highest reliability was observed with ICC(3,k) at 0.95 (95% CI: 0.93–0.96), confirming that the composite scores derived from multiple evaluators provide robust and consistent assessments. These findings support the methodological rigor of our AGREE-II evaluations and reinforce the reliability of guideline quality assessments. Linear regression demonstrated that newer guidelines were of higher quality (r^2^ = 0.17, 95% CI: 0.11–0.33; p = 0.036; Fig. 4). There was no difference in quality between guidelines with and without professional body endorsement (median: 68% vs 67%).

## Discussion

This scoping review and quality assessment includes 22 CPGs reporting recommendations on diagnosis and management of adult patients with acute pancreatitis. It highlights the lack of comprehensive coverage of these CPGs across the entire patient pathway—with disproportionately lower recommendations supporting recovery and rehabilitation after acute pancreatitis. The overall quality of CPGs has improved over the past decade, reflecting a growing emphasis on evidence-based care. However, substantial discrepancies remain between the strength of recommendations and the quality of evidence underpinning them. For instance, while nearly half of all recommendations were categorised as strong, only 16.7% were supported by high-level evidence. This misalignment highlights the ongoing need for more robust research to substantiate clinical guidelines in AP.

There is a lack of guidance within CPGs on recovery and rehabilitation in patients with AP. Of the 718 recommendations extracted from guidelines in this study, only 19 (2.6%) related to recovery or rehabilitation. The quality of evidence within these recommendations is also low with only 37.8% (n = 7/19) supported by high level evidence. This is especially important with the growing emphasis towards universal health coverage–from presentation to recovery.^13^ Whilst in-hospital management received considerable attention for research, post-discharge care (including recovery, rehabilitation, and prevention) was poorly addressed. Given the risk of recurrence, as well as potential long term complications of AP such as type 3c diabetes, this represents a critical gap in the current CPGs. Furthermore, the impact on psychological wellbeing cannot be neglected given one-in-two patients report depression or anxiety at follow-up.^31^ This highlights the urgent need for research focused on the recovery phase in AP. Its importance has been emphasised in a recent consensus process involving hundreds of clinicians and patients to identify future research priorities.^9^ Therefore, evaluation through research will inform clinical practice as we seek to achieve universal health coverage and sustainable developments goal 3 by 2030.

Further, there were no recommendations highlighted in the palliation domain across the different CPGs. Although AP is often considered a self-limiting condition, patients with severe or necrotising disease may experience a high symptom burden, prolonged hospital stays, and significant morbidity. In such cases, a palliative approach that is focussed on symptom management, quality of life, and supportive care may be needed. For instance, pain management remains a cornerstone of treatment, particularly in patients with persistent abdominal pain despite resolution of the acute episode. In addition to opioids and adjunctive analgesics, multimodal pain relief strategies, including regional anaesthesia and non-pharmacological interventions, should be considered. Nutritional support also plays a role in palliation, especially for patients who develop pancreatic insufficiency or experience recurrent AP episodes that impact their nutritional status. Early enteral feeding is recommended in severe cases, but in patients with long-term complications, dietary modifications and enzyme supplementation may be required to maintain nutritional balance and prevent weight loss. In rare cases where acute pancreatitis progresses to multisystem organ failure, discussions regarding goals of care and end-of-life decision-making may be necessary. For patients with refractory AP or those at high risk of mortality, palliative care services can provide holistic support, addressing not only symptom relief but also psychological and family-centred concerns. While palliation is not a primary focus of AP management, incorporating symptom-based supportive care principles in severe or chronic cases may improve overall patient well-being. Further research is needed to explore the role of palliative care in AP, particularly in patients with recurrent or treatment-resistant disease.

The majority of recommendations exist for in-hospital management or treatment. Despite this, historical shifts in recommendations for fluid resuscitation and antibiotic use offer a compelling case study illustrating guideline variability driven by evolving evidence. One of the most debated areas is early fluid resuscitation. Earlier guidelines often recommended aggressive fluid resuscitation to prevent hypoperfusion-related pancreatic necrosis; however, more recent evidence suggests that excessive fluid administration may contribute to complications such as abdominal compartment syndrome and increased mortality.^32^ As a result, some recent guidelines advocate for a more conservative, goal-directed fluid resuscitation strategy. Similarly, the use of prophylactic antibiotics in AP remains controversial. Initial recommendations favoured broad antibiotic use to prevent infected pancreatic necrosis, but subsequent randomised controlled trials failed to demonstrate a mortality benefit. Consequently, more recent guidelines recommend selective antibiotic use based on clinical deterioration rather than routine prophylaxis.^33^, ^34^, ^35^ These changes highlight the importance of continuously updating guidelines to reflect the latest evidence, ensuring that recommendations are both safe and effective.

There was a disconnect between the strength of recommendations and the quality of evidence supporting them. Many of the guidelines recommend strong actions, yet the quality of the underlying evidence is often only moderate or low. This is particularly concerning for in-hospital management, where the majority of strong recommendations (48.4%, n = 337/696) are not evidenced by high-quality evidence. For clinicians, this creates a challenge in balancing guideline adherence with clinical judgment, especially in the absence of robust supporting data. A recent national cohort study highlighted variable adherence to evidence-based practice across 2580 patients in the United Kingdom.^7^ Discrepancies between the strength of recommendations and the underlying evidence quality is not unique to guidelines on acute pancreatitis, but is seen across various medical fields, including sepsis and critical care medicine. Common reasons they vary include reliance on expert consensus, poor methodology used in its development, and absence of high-quality randomised trial. Therefore, future guideline development should incorporate more robust methodologies, such as adaptive trial designs and real-world evidence, to strengthen the reliability of recommendations. Policymakers and researchers must focus on generating high-quality evidence to strengthen the foundation of these recommendations.

It is important to acknowledge that the identified gaps in the current clinical practice guidelines (CPGs) for acute pancreatitis are not merely documentation oversights but represent genuine, impactful clinical problems. Gaps in CPGs means mean that patients are likely underserved in certain clinical domains, leading to poorer health outcomes and inefficient resource use within health systems worldwide.

First, limited guidance on crucial aspects of care, such as rehabilitation and structured follow-up, contributes directly to poorer patient outcomes, including increased risks of recurrence, higher rates of hospital readmission, and diminished quality of life post-discharge. Recent observational studies^7^^,^^19^ highlight the severity of these omissions, showing clear links between inconsistent follow-up care, inadequate rehabilitation services, and poor clinical outcomes and resource utilisation. Second, a lack of standardised guidance on preventive interventions such as alcohol cessation, smoking cessation, and obesity management likely contributes to ongoing patient morbidity and recurrence of acute pancreatitis episodes.^36^, ^37^, ^38^ The clinical effectiveness of these preventive measures is well-established, yet their inconsistent integration into CPGs highlights a significant missed opportunity to improve patient outcomes. Third, variable uptake or implementation of best evidence for fluid resuscitation and antibiotic use have been associated with differences in hospital length of stay, complication rates, and overall morbidity.^7^^,^^9^^,^^19^^,^^32^ Consequently, our findings justify a meaningful shift in future guideline development towards a more comprehensive, patient-centred approach that explicitly addresses these gaps. Future guidelines must integrate robust evidence-based recommendations on rehabilitation, preventive strategies, and structured long-term follow-up. To achieve this, there is a pressing need for targeted clinical and implementation research to systematically quantify the benefits of addressing these guideline gaps, thereby ensuring future guidelines are clinically meaningful, actionable, and globally applicable. Addressing these gaps through evidence-based guideline harmonisation could improve patient outcomes and enhance healthcare resource efficiency. By minimising inconsistencies in AP management, there is an opportunity to improve the quality of care, reduce unnecessary hospitalisations, and optimise healthcare expenditures.

Our study demonstrated notable disparities in the development of CPGs, where majority (77.3%) originated from HICs. This may reflect greater research infrastructure, resource availability, and capacity to perform high-quality clinical trials. This uneven distribution significantly influences the generalisability of recommendations, as CPGs from resource-rich settings often assume the availability of advanced healthcare services and technologies. For example, recommendations around conservative fluid resuscitation strategies and selective antibiotic use rely heavily on advanced diagnostic and monitoring facilities more readily accessible in HICs. By contrast, limited recommendations from LMICs highlight challenges in evidence generation, implementation capacity, and health system resource limitations, rather than solely a lack of clinical relevance. Consequently, recommendations applicable to LMIC settings often default to expert consensus rather than rigorous clinical trial data, which may limit their reliability and applicability.

This study's strengths lie in its comprehensive scope and the rigorous methodology employed. By focusing on multiple facets of CPGs, including the strength and quality of recommendations, we provide a thorough evaluation of current guidelines. Additionally, the use of a patient and public involvement (PPI) group ensures that our findings are relevant and meaningful to those with lived experience of the disease. However, several limitations should be noted. First, the exclusion of non-English language guidelines may limit the generalisability of the findings, particularly for non-English-speaking regions where the burden of acute pancreatitis is high. Second, while this review highlights gaps in recovery recommendations, it does not delve into the specific barriers to implementing these recommendations in clinical practice, for example disparities in regional service provision. Third, a potential methodological limitation of our study is related to the use of the AGREE-II instrument, published in 2017. Evaluators' awareness of the publication date and the standards introduced by AGREE-II could have introduced unintentional bias, potentially favouring guidelines published after 2017 that inherently align more closely with current reporting standards. Consequently, earlier guidelines might have scored lower due to reporting styles and expectations prevalent at the time rather than their actual methodological rigor. To mitigate this bias, we employed multiple independent evaluators, rigorous training, and calibration to ensure scoring consistency. Despite these measures, the influence of temporal reporting bias cannot be eliminated completely. Nevertheless, our analysis of guideline quality over time offers an important benchmark for future evaluations and highlights areas for improvement in the ongoing development of clinical practice guidelines for acute pancreatitis and other clinical conditions. Fourth, most included guidelines are developed in high income countries, which may limit the applicability to low- and middle-income countries, where resource constraints, diagnostic capacity, and treatment access differ significantly. Therefore, secondary analysis of characteristics of CPGs by country income were not possible. However, future efforts should focus on developing context-specific recommendations tailored to LMIC settings, incorporating considerations such as cost-effectiveness, availability of interventions, and feasibility of implementation. Fifth, we did not collect data on key clinical outcomes such as post-AP mortality, morbidity, recurrence rates and long-term complications since such data are not routinely reported as part of CPGs. However, this highlights for better reporting to ensure that are clear parallels on how different recommendations may improve particular outcomes for these patients. Finally, the reliance on published CPGs may overlook informal or unpublished guidelines that could influence clinical decision-making in different healthcare systems. Further, we have not evaluated the direction of individual recommendations within the same domain, as these may also likely to vary by time of publication and available evidence at that point in time.

The findings from this scoping review and quality assessment have important implications for both policy and future research. First, future research efforts in pancreatitis should focus on addressing domains where limited evidence was identified. For instance, research is required to explore effective recovery strategies, including rehabilitation protocols and preventive measures, are essential. Further, prioritisation of clinical trials targeting areas of significant variability, such as fluid management, antibiotic use, and long-term monitoring strategies is much needed (Table 3). Second, healthcare systems, policymakers, and special interest groups must prioritise the development of more comprehensive guidelines that address the entire patient care continuum, including recovery and rehabilitation. These areas are critical for improving long-term outcomes, reducing recurrence, and enhancing quality of life for patients. Third, stronger collaboration between multidisciplinary teams, including gastroenterologists, surgeons, nurses, allied healthcare professionals, rehabilitation specialists, and policymakers, is needed to ensure that guidelines are not only evidence-based but also equitable and applicable across diverse healthcare settings. Therefore, clear actionable targets for clinicians, policymakers and researchers include: (i) development of an internationally harmonised guideline synthesis to provide standardised, evidence-based recommendations; and (ii) Integration of real-world data and implementation science to bridge the gap between guideline recommendations and clinical practice (Table 4).

**Table 3:** Summary of key domains and future research questions in acute pancreatitis.

Indicators by domains	Relevant future research questions
Promotion & prevention	
Alcohol cessation	• How can alcohol cessation services be integrated into current pathways for patients with acute pancreatitis? • What is the clinical effectiveness of embedding alcohol cessation pathways into patients with acute pancreatitis?
Smoking cessation	• How can smoking cessation services be integrated into current pathways for patients with acute pancreatitis? • What is the clinical effectiveness of embedding smoking cessation pathways into patients with acute pancreatitis?
Weight reduction or obesity management	• What weight or obesity management interventions or services be integrated into current pathways for patients with acute pancreatitis? • What is the clinical effectiveness of embedding weight or obesity management interventions or services into patients with acute pancreatitis?
Treatment	
Diagnosis of disease	None
Assessment of severity	• What are the best prognostic tools for risk assessing severity of acute pancreatitis?
Early in-hospital management	• How can evidence-based practice for early (i.e., within 48 h) from admission be implemented? • What is the clinical-effectiveness of biologics in reducing progression or severity of acute pancreatitis?
Late in-hospital management	None
Interventional management	None
Intensive care management	None
Organisation or model of care	• What are the best models of care the management of patients with complex acute pancreatitis?
Rehabilitation	
Follow-up care	• What interventions should form part of routine follow-up in patients with acute pancreatitis? • What is the clinical effectiveness of a structured follow-up in reducing readmission and improve quality of life? • What are the core outcomes to measure follow-up successfully?
Diagnosis and management of new long-term conditions	• What is the prevalence of new long-term conditions in patients with new onset acute pancreatitis?
Palliation	
Pain management	• What is the best pain management in patients with complex acute pancreatitis or long-term pain?

**Table 4:** Key actionable areas and plan for clinicians, researchers and policymakers for acute pancreatitis.

**For clinicians**	
Evidence-based implementation strategies	• Develop targeted educational and training programs focusing specifically on evidence-based guideline adherence for critical clinical practices identified as variable (e.g., fluid resuscitation strategies, antibiotic stewardship). • Implement audit and feedback systems to systematically evaluate adherence to recommendations, particularly in early in-hospital management.
Enhance recovery pathways	• Consider standardised protocols to integrate routine recovery and rehabilitation assessment into discharge planning, specifically addressing follow-up care to manage psychological wellbeing and long-term complications, including exocrine insufficiency. • Establish multidisciplinary teams involving gastroenterologists, surgeons, rehabilitation specialists, and psychologists to support a holistic approach to patient management post-discharge.
**For policymakers**	
Guideline harmonisation and equity	• Initiate international collaboration to establish minimum essential guidelines for acute pancreatitis management, emphasising equitable coverage across high-income countries (HICs) and low- and middle-income countries (LMICs). • Develop and support regional guideline adaptations explicitly tailored to LMICs, incorporating practical considerations of cost-effectiveness, healthcare infrastructure, and local disease burden.
Promotion of recovery and rehabilitation	• Incentivize and support national healthcare bodies to include comprehensive recovery and rehabilitation measures within existing clinical guidelines. • Allocate dedicated resources and funding for research initiatives aimed at evaluating the clinical impact and cost-effectiveness of post-acute pancreatitis rehabilitation programs.
**For researchers**	
Prioritising development of high-quality research	• Prioritise high-quality randomized controlled trials and real-world studies specifically targeting controversial areas identified in the review, such as optimal fluid resuscitation volumes, selective antibiotic protocols, and effective pain management strategies. • Encourage the development of international registries and collaborative databases to monitor adherence to guidelines, patient outcomes, and healthcare resource utilization across different healthcare settings globally.
Context-specific research in LMICs	• Conduct pragmatic trials and implementation research specifically tailored to resource-constrained environments, identifying practical interventions that optimise care in these settings. • Study the barriers to guideline adoption, including healthcare infrastructure limitations, cultural acceptability, and economic feasibility, ensuring findings are actionable and contextually relevant
Prioritise critical evidence gaps	• Explore optimal approaches to recovery or rehabilitation in patients following an episode of acute pancreatitis. • Explore the potential role of palliative care interventions for patients experiencing severe acute pancreatitis or significant symptom burden, to understand its impact on quality of life and healthcare costs.
Health economic analysis	• Undertake rapid and high-quality health economic analysis to contextually understand impact of inefficiencies of current pathways to health systems.

Note that these actionable areas are based on a rapid scoping review and discussion with the study management group, based on a qualitative summary.

This scoping review and quality assessment highlights both progress and persistent gaps in the development of clinical practice guidelines for the management of acute pancreatitis. While the quality of CPGs has improved, significant discrepancies exist between the strength of recommendations and the quality of evidence. Moreover, recovery and rehabilitation are underrepresented areas, and there is a pressing need for more comprehensive guidance in these domains. Addressing these gaps will require concerted efforts from researchers, clinicians, and policymakers to ensure that all patients with acute pancreatitis receive high-quality, equitable care throughout their treatment journey, from diagnosis to recovery.

## Contributors

SKK, VG and ML contributed to data curation and interpretation. SKK and MJL contributed to data analysis. The writing group contributed to writing and critical revision of the manuscript. SKK, and MJL, verified the underlying data in the study. The corresponding author had final responsibility for the decision to submit for publication.

## Data sharing statement

Data sharing requests will be considered by the writing group upon written request to the corresponding author. De-identified participant data and/or other pre-specified data will be available, subject to a written proposal and an agreed data sharing agreement.

## Declaration of interests

All authors declare no conflict of interest.
