# Supplementary material for: Gaps and uncertainties in the management of acute pancreatitis: a scoping review and quality assessment of clinical practice guidelines
Source: eClinicalMedicine. 2025 May 15;84:103216. doi: 10.1016/j.eclinm.2025.103216 (PMC12273737; doi:10.1016/j.eclinm.2025.103216)
Supplement: Supplementary Figures and Tables [file mmc1.docx]

**Gaps and uncertainties in the management of pancreatitis: A scoping review of clinical practice guidelines**

Sivesh K Kamarajah, MBChB^1, 2^, Vignesh Gopalan, MBChB^3^, Zarnigar Khan, MBChB ^4^, Daniel M Baker, MBChB ^5^, Amy Lucas^6^, David Hawkins^6^, Stacey Munnelly, RGN^7^, Marianne Hollyman, MD^3^, Laura Magill^1^, Matthew J Lee, PhD^1^

1. Department of Applied Health Sciences, School of Health Sciences, College of Medicine and Health, University of Birmingham, Birmingham, United Kingdom
2. NIHR Global Health Research Unit on Global Surgery, University of Birmingham, Birmingham, United Kingdom
3. Somerset NHS Foundation Trust, Musgrove Park Hospital, Taunton, United Kingdom.
4. Department of Surgery, Sheffield Teaching Hospitals NHS Foundation Trust, Sheffield, United Kingdom
5. Department of Surgery, Leeds Teaching Hospitals NHS Foundation Trust, Leeds, United Kingdom
6. GUTS UK Charity, London
7. Department of Gastroenterology, Manchester Royal Infirmary, Manchester University NHS Foundation Trust, Manchester, United Kingdom.

**Corresponding author**: Mr Sivesh Kathir Kamarajah, NIHR Doctoral Fellow, NIHR Global Health Research Unit on Global Surgery, Institute of Applied Health Research, University of Birmingham, Birmingham, United Kingdom. Email: s.k.kamarajah@bham.ac.uk

**Word count:**

**Abstract word count:**378

**Keywords:** pancreatitis; universal health coverage; rehabilitation

**Short title:** Uncertainties in pancreatitis

**Conflict of interest:** There are no conflicts of interest to declare.

**Funding:** SK was funded by the NIHR Doctoral Research Fellowship (NIHR303288). The views expressed in this publication are those of the author(s) and not necessarily those of the NIHR or the UK government. The funders had no role in study design, data collection, data analysis, data interpretation, writing of the report, or the decision to submit the paper for publication.

**Data sharing:** Data sharing requests will be considered by the writing group upon written request to the corresponding author.

Table of Contents

[Table S1. Preferred Reporting Items for Systematic reviews and Meta-Analyses extension for Systematc Reviews (PRISMA) Checklist 3](#_Toc194261460)

[Figure S1. Summary of key processes and people involved in different stages of the scoping review. 6](#_Toc194261461)

[Table S2. Summary of search terms used for the scoping review 7](#_Toc194261462)

[Table S3. Summary of the data extraction sheet for the included studies in the scoping review 8](#_Toc194261463)

[Table S4. Adapted UHC effective coverage measurement framework, according to the health service types domain 9](#_Toc194261464)

[Table S5. Details of the 12 indicators for assessing coverage of clinical practice guidelines for people with acute pancreatitis, by health service type 10](#_Toc194261465)

[Figure S2. Differences in strength of recommendations and quality of evidence reported within clinical practice guidelines included in the systematic review 11](#_Toc194261466)

[Table S6. Number of recommendations by strength within each indicator, grouped by key domains from the clinical practice guidelines 12](#_Toc194261467)

[Table S7. Number of recommendations by quality within each indicator, grouped by key domains from the clinical practice guidelines 13](#_Toc194261468)

[Table S8. Study characteristics by presence of professionals with expertise in gastroenterology 14](#_Toc194261469)

[Table S9. Summary of the AGREE-II instruments by items across the different reviewers and average scores for each reviewer 15](#_Toc194261470)

# **Table S1.** Preferred Reporting Items for Systematic reviews and Meta-Analyses extension for Systematc Reviews (PRISMA) Checklist

| **Section and Topic** | **Item #** | **Checklist item** | **Location where item is reported** |
| --- | --- | --- | --- |
| **TITLE** | | |  |
| Title | 1 | Identify the report as a systematic review. | 1 |
| **ABSTRACT** | | |  |
| Abstract | 2 | See the PRISMA 2020 for Abstracts checklist. | 2 |
| **INTRODUCTION** | | |  |
| Rationale | 3 | Describe the rationale for the review in the context of existing knowledge. | 3 |
| Objectives | 4 | Provide an explicit statement of the objective(s) or question(s) the review addresses. | 3-4 |
| **METHODS** | | |  |
| Eligibility criteria | 5 | Specify the inclusion and exclusion criteria for the review and how studies were grouped for the syntheses. | 5 |
| Information sources | 6 | Specify all databases, registers, websites, organisations, reference lists and other sources searched or consulted to identify studies. Specify the date when each source was last searched or consulted. | 5 |
| Search strategy | 7 | Present the full search strategies for all databases, registers and websites, including any filters and limits used. | 5 |
| Selection process | 8 | Specify the methods used to decide whether a study met the inclusion criteria of the review, including how many reviewers screened each record and each report retrieved, whether they worked independently, and if applicable, details of automation tools used in the process. | 5 |
| Data collection process | 9 | Specify the methods used to collect data from reports, including how many reviewers collected data from each report, whether they worked independently, any processes for obtaining or confirming data from study investigators, and if applicable, details of automation tools used in the process. | 5-6 |
| Data items | 10a | List and define all outcomes for which data were sought. Specify whether all results that were compatible with each outcome domain in each study were sought (e.g. for all measures, time points, analyses), and if not, the methods used to decide which results to collect. | 7 |
|  | 10b | List and define all other variables for which data were sought (e.g. participant and intervention characteristics, funding sources). Describe any assumptions made about any missing or unclear information. | 7 |
| Study risk of bias assessment | 11 | Specify the methods used to assess risk of bias in the included studies, including details of the tool(s) used, how many reviewers assessed each study and whether they worked independently, and if applicable, details of automation tools used in the process. | 7-8 |
| Effect measures | 12 | Specify for each outcome the effect measure(s) (e.g. risk ratio, mean difference) used in the synthesis or presentation of results. | 8-9 |
| Synthesis methods | 13a | Describe the processes used to decide which studies were eligible for each synthesis (e.g. tabulating the study intervention characteristics and comparing against the planned groups for each synthesis (item #5)). | 8-9 |
|  | 13b | Describe any methods required to prepare the data for presentation or synthesis, such as handling of missing summary statistics, or data conversions. | 8-9 |
|  | 13c | Describe any methods used to tabulate or visually display results of individual studies and syntheses. | 8-9 |
|  | 13d | Describe any methods used to synthesize results and provide a rationale for the choice(s). If meta-analysis was performed, describe the model(s), method(s) to identify the presence and extent of statistical heterogeneity, and software package(s) used. | 8-9 |
|  | 13e | Describe any methods used to explore possible causes of heterogeneity among study results (e.g. subgroup analysis, meta-regression). | 8-9 |
|  | 13f | Describe any sensitivity analyses conducted to assess robustness of the synthesized results. | 8-9 |
| Reporting bias assessment | 14 | Describe any methods used to assess risk of bias due to missing results in a synthesis (arising from reporting biases). | 8-9 |
| Certainty assessment | 15 | Describe any methods used to assess certainty (or confidence) in the body of evidence for an outcome. | 8-9 |
| **RESULTS** | | |  |
| Study selection | 16a | Describe the results of the search and selection process, from the number of records identified in the search to the number of studies included in the review, ideally using a flow diagram. | 10 |
|  | 16b | Cite studies that might appear to meet the inclusion criteria, but which were excluded, and explain why they were excluded. | 10 |
| Study characteristics | 17 | Cite each included study and present its characteristics. | 10 |
| Risk of bias in studies | 18 | Present assessments of risk of bias for each included study. | 10 |
| Results of individual studies | 19 | For all outcomes, present, for each study: (a) summary statistics for each group (where appropriate) and (b) an effect estimate and its precision (e.g. confidence/credible interval), ideally using structured tables or plots. | 10 |
| Results of syntheses | 20a | For each synthesis, briefly summarise the characteristics and risk of bias among contributing studies. | 10 |
|  | 20b | Present results of all statistical syntheses conducted. If meta-analysis was done, present for each the summary estimate and its precision (e.g. confidence/credible interval) and measures of statistical heterogeneity. If comparing groups, describe the direction of the effect. | 10 |
|  | 20c | Present results of all investigations of possible causes of heterogeneity among study results. | 10 |
|  | 20d | Present results of all sensitivity analyses conducted to assess the robustness of the synthesized results. | 11 |
| Reporting biases | 21 | Present assessments of risk of bias due to missing results (arising from reporting biases) for each synthesis assessed. | 11 |
| Certainty of evidence | 22 | Present assessments of certainty (or confidence) in the body of evidence for each outcome assessed. | 11 |
| **DISCUSSION** | | |  |
| Discussion | 23a | Provide a general interpretation of the results in the context of other evidence. | 12-15 |
|  | 23b | Discuss any limitations of the evidence included in the review. | 15 |
|  | 23c | Discuss any limitations of the review processes used. | 15 |
|  | 23d | Discuss implications of the results for practice, policy, and future research. | 16 |
| **OTHER INFORMATION** | | |  |
| Registration and protocol | 24a | Provide registration information for the review, including register name and registration number, or state that the review was not registered. | Not registered |
|  | 24b | Indicate where the review protocol can be accessed, or state that a protocol was not prepared. | Not registered |
|  | 24c | Describe and explain any amendments to information provided at registration or in the protocol. | Not registered |
| Support | 25 | Describe sources of financial or non-financial support for the review, and the role of the funders or sponsors in the review. | 1 |
| Competing interests | 26 | Declare any competing interests of review authors. | 1 |
| Availability of data, code and other materials | 27 | Report which of the following are publicly available and where they can be found: template data collection forms; data extracted from included studies; data used for all analyses; analytic code; any other materials used in the review. | 1 |

# **Figure S1.** Summary of key processes and people involved in different stages of the scoping review.

# **Table S2.** Summary of search terms used for the scoping review

| **No.** | **Terms** |
| --- | --- |
| 1 | exp pancreatitis/ or pancreatitis.ti,ab. or exp pancreatitis, alcoholic/ |
| 2 | acute pancreatitis.ti,ab. |
| 3 | 1 or 2 |
| 4 | guidleline.pt. or exp guideline/ or exp practice guideline/ or exp Consensus/ or exp Consensus Development Conference, NIH/ or exp Consensus Development Conference/ or (consensuses or consensus or position statement or position statements or practice parameter or practice parameters or "appropriate use criteria" or appropriateness criteria or guidance statement or guidance statements or guideline or guidelines or bulletin).ti,bt. |
| 5 | 3 or 4 |
|  |  |

# **Table S3.** Summary of the data extraction sheet for the included studies in the scoping review

| **Category** | **Definition** |
| --- | --- |
| **Study characteristics** | |
| Study name | Includes author name and year |
| Study year | Study starts and end date |
| Country income | High income / Low- or middle-income defined according to World Bank group |
| Country | This is defined as the countries in which the study was delivered. |
| **Guideline development process** | |
| People involved in the development | This refers to the specialty or expertise involved in developing the clinical practice guidelines |
| Endorsement | This refers to the professional society or organisation by which the |
| **Guideline content** | |
| Key domains with the guideline | This was defined through a consensus process to align them according to the principles of Universal Health Coverage effective coverage framework |
| Indicators for recommendation | This was defined by the recommendations under the relevant indicators within each domain included in the clinical practice guidelines. |
| Strength of recommendation | Strength was defined as strong, moderate, weak, or very weak, where available. If grading of strength of the recommendation not provided, this was defined as not reported. |
| Quality of recommendation | Quality of evidence underpinning each recommendation was defined as high, moderate, low, or very low, where available. If grading of evidence was not provided, this was defined as not reported. |

# **Table S4.** Adapted UHC effective coverage measurement framework, according to the health service types domain

| **Health Service type** | | | | |
| --- | --- | --- | --- | --- |
| **Promotion** | **Prevention** | **Treatment** | **Rehabilitation** | **Palliation** |
| Smoking cessation service | Smoking cessation service | Diagnosis of disease | Follow-up care |  |
| Alcohol cessation | Alcohol cessation | Assessment of severity | Diagnosis and management of long-term condition |  |
| Weight reduction or obesity management | Weight reduction or obesity management | Early in-hospital management |  |  |
|  |  | Late in-hospital management |  |  |
|  |  | Interventional management |  |  |
|  |  | Intensive care unit management |  |  |
|  |  | Organisation or model of care* |  |  |

*Model of care, according to the WHO UHC conceptual framework is defined as Selection and planning of services, Service design, organisation and facility management and community linkages and engagement

# **Table S5.** Details of the 12 indicators for assessing coverage of clinical practice guidelines for people with acute pancreatitis, by health service type

| **Domains** | **Indicators** | **Rationale** |
| --- | --- | --- |
| Prevention; Promotion | Smoking cessation service | Tobacco smoking is a risk factor for pancreatitis. Identifying current smokers with clear guidance or pathway for reducing risk of public or patients for risk of pancreatitis. |
| Prevention; Promotion | Alcohol cessation | Alcohol is a major risk factor for pancreatitis and progression of disease. Recommendations covering alcohol cessation is important in the prevention of this disease and recurrence of disease. These may include screening of patients using AUDIT-C or management. |
| Prevention; Promotion | Weight reduction or obesity management | Obesity is a major risk factor for gallstone disease that can cause gallstone-related acute pancreatitis. Reducing weight or strategies to support this can reduce risk of disease and recurrence. |
| Treatment | Diagnosis of disease | Timely diagnosis is important to prevent progression of acute pancreatitis. |
| Treatment | Assessment of severity | Assessment of severity is defined as the use of risk stratification measures, either objective or subjective approaches. |
| Treatment | Early in-hospital management | Early management is defined as provision or guidance which includes (not exhaustive) pain management, fluid control, nutrition, and antibiotics within 48 hours to 72 hours from admission. |
| Treatment | Late in-hospital management | Late management is defined as provision or guidance which includes (not exhaustive) management of complications related to pancreatitis such as pseudocyst, collection and pancreatic necrosis. |
| Treatment | Interventional management | Interventional management includes the use of radiology, endoscopic interventions or surgery in the diagnosis and/or treatment of complications related to acute pancreatitis. |
| Treatment | Intensive care unit management | This is defined as the guidance around the use of intensive care unit and criteria for escalation in patients with acute pancreatitis. |
| Treatment | Organisation or model of care | This is defined as the guidance around the organisation of service delivery including different team members, tertiary and secondary servies and best processes to achieve this. |
| Rehabilitation | Follow-up care | This is defined as post-discharge care involving guidance on appointments to help with recovery of patients. |
| Rehabilitation | Diagnosis and management of long-term condition | Patients with acute pancreatitis are at risk of developing physical conditions (i.e., type 3c diabetes, malnutrition) and mental conditions (i.e., anxiety and depression). This indicator refers to the guidance provided on what healthcare professionals should do to help diagnose and manage these conditions. |

# **Figure S2.** Differences in strength of recommendations and quality of evidence reported within clinical practice guidelines included in the systematic review

# **Table S6.** Number of recommendations by strength within each indicator, grouped by key domains from the clinical practice guidelines

| **Key domains and subdomains** | **Number, n (%)** | **Strong** | **Moderate** | **Weak** | **Very weak** | **Not reported** |
| --- | --- | --- | --- | --- | --- | --- |
| **Promotion** | **0** | **-** | **-** | **-** | **-** | **-** |
| **Prevention** | **3** | **0 (0)** | **0 (0)** | **1 (33.3)** | **0 (0)** | **2 (66.7)** |
| Alcohol cessation | 2 (66.7) | 0 (0) | 0 (0) | 1 (50) | 0 (0) | 1 (50) |
| Smoking cessation | 1 (33.3) | 0 (0) | 0 (0) | 0 (0) | 0 (0) | 1 (100) |
| **Treatment** | **696** | **337 (48.4)** | **50 (7.2)** | **176 (25.3)** | **35 (5)** | **98 (14.1)** |
| Diagnosis of disease | 159 (22.8) | 67 (42.1) | 12 (7.5) | 36 (22.6) | 14 (8.8) | 30 (18.9) |
| Assessment of severity | 48 (6.9) | 19 (39.6) | 4 (8.3) | 14 (29.2) | 0 (0) | 11 (22.9) |
| Early in-hospital management | 215 (30.9) | 114 (53.0) | 8 (3.7) | 53 (24.7) | 10 (4.7) | 30 (14) |
| Late in-hospital management | 123 (17.7) | 58 (47.2) | 16 (13) | 32 (26) | 4 (3.3) | 13 (10.6) |
| Interventional management | 113 (16.2) | 59 (52.2) | 9 (8) | 32 (28.3) | 2 (1.8) | 11 (9.7) |
| Intensive care management | 34 (4.9) | 18 (52.9) | 0 (0) | 8 (23.5) | 5 (14.7) | 3 (8.8) |
| Organisation or model of care | 4 (0.6) | 2 (50.0) | 1 (25) | 1 (25) | 0 (0) | 0 (0) |
| **Rehabilitation** | **19** | **11 (57.9)** | **3 (15.8)** | **2 (10.5)** | **0 (0)** | **3 (15.8)** |
| Follow-up care | 17 (89.5) | 11 (64.7) | 2 (11.8) | 1 (5.9) | 0 (0) | 3 (17.6) |
| Diagnosis and management of new long-term conditions | 2 (10.5) | 0 (0) | 1 (50) | 1 (50) | 0 (0) | 0 (0) |
| **Palliation** | **0** | **-** | **-** | **-** | **-** | **-** |

# **Table S7.** Number of recommendations by quality within each indicator, grouped by key domains from the clinical practice guidelines

| **Key domains and subdomains** | **Number, n (%)** | **High** | **Moderate** | **Low** | **Very low** | **Not reported** |
| --- | --- | --- | --- | --- | --- | --- |
| **Promotion** | **0** | **-** | **-** | **-** | **-** | **-** |
| **Prevention** | **3** | **0 (0)** | **0 (0)** | **1 (33.3)** | **0 (0)** | **2 (66.7)** |
| Alcohol cessation | 2 (66.7) | 0 (0) | 0 (0) | 0 (0) | 1 (50) | 1 (50) |
| Smoking cessation | 1 (33.3) | 0 (0) | 0 (0) | 0 (0) | 0 (0) | 1 (100) |
| **Treatment** | **696** | **125 (17.9)** | **250 (35.9)** | **155 (22.2)** | **68 (9.8)** | **99 (14.2)** |
| Diagnosis of disease | 159 (22.8) | 22 (13.8) | 44 (27.7) | 25 (15.7) | 38 (23.9) | 30 (18.9) |
| Assessment of severity | 48 (6.9) | 4 (8.3) | 23 (47.9) | 0 (0) | 10 (20.8) | 11 (22.9) |
| Early in-hospital management | 215 (30.9) | 55 (25.6) | 74 (34.4) | 17 (7.9) | 39 (18.1) | 30 (14) |
| Late in-hospital management | 123 (17.7) | 12 (9.8) | 55 (44.7) | 12 (9.8) | 31 (25.2) | 13 (10.6) |
| Interventional management | 113 (16.2) | 25 (21.9) | 47 (41.2) | 8 (7) | 22 (19.3) | 1**1** (10.5) |
| Intensive care management | 34 (4.9) | 7 (20.6) | 6 (17.6) | 6 (17.6) | 12 (35.3) | 3 (8.8) |
| Organisation or model of care | 4 (0.6) | 0 (0) | 1 (25) | 0 (0) | 3 (75) | 0 (0) |
| **Rehabilitation** | **19** | **7 (36.8)** | **6 (31.6)** | **3 (15.8)** | **0 (0)** | **3 (15.8)** |
| Follow-up care | 17 (89.5) | 7 (41.2) | 4 (23.5) | 0 (0) | 3 (17.6) | 3 (17.6) |
| Diagnosis and management of new long-term conditions | 2 (10.5) | 0 (0) | 2 (100) | 0 (0) | 0 (0) | 0 (0) |
| **Palliation** | **0** | **-** | **-** | **-** | **-** | **-** |

# **Table S8.** Study characteristics by presence of professionals with expertise in gastroenterology

|  |  | **No** | **Yes** | **Total** | **p-value** |
| --- | --- | --- | --- | --- | --- |
| Total N (%) |  | 5 (22.7) | 17 (77.3) | 22 |  |
| Published year | 2001 - 2010 | 1 (20.0) | 6 (35.3) | 7 (31.8) | 0.808 |
|  | 2011-2020 | 3 (60.0) | 8 (47.1) | 11 (50.0) |  |
|  | 2021-2024 | 1 (20.0) | 3 (17.6) | 4 (18.2) |  |
| MDT, n | Median (IQR) | 1.0 (1.0 to 2.0) | 3.0 (2.0 to 4.0) | 2.0 (1.0 to 3.8) | 0.091 |
| Surgery |  | 2 (40.0) | 10 (58.8) | 12 (54.5) | 0.816 |
| Pancreatologists |  | 4 (80.0) | 4 (23.5) | 8 (36.4) | 0.075 |
| Emergency care |  | 0 (0.0) | 3 (17.6) | 3 (13.6) | 0.788 |
| Critical care |  | 0 (0.0) | 4 (23.5) | 4 (18.2) | 0.589 |
| Anaesthetists |  | 0 (0.0) | 3 (17.6) | 3 (13.6) | 0.788 |
| Dietician |  | 1 (20.0) | 2 (11.8) | 3 (13.6) | 1.000 |
| Radiology |  | 0 (0.0) | 6 (35.3) | 6 (27.3) | 0.324 |
| Rehabilitation |  | 0 (0.0) | 1 (5.9) | 1 (4.5) | 1.000 |
| Policymaker |  | 1 (20.0) | 0 (0.0) | 1 (4.5) | 0.505 |
| Type of guideline | CPG | 4 (80.0) | 9 (75.0) | 13 (76.5) | 0.534 |
|  | Consensus statement | 1 (20.0) | 1 (8.3) | 2 (11.8) |  |
|  | Position Statement |  | 2 (16.7) | 2 (11.8) |  |
| Country income | High & LMIC | 1 (20.0) |  | 1 (4.5) | 0.247 |
|  | LMIC |  | 1 (5.9) | 1 (4.5) |  |
|  | Not reported | 1 (20.0) | 2 (11.8) | 3 (13.6) |  |
| **Guideline domain** |  |  |  |  |  |
| Prevention | yes | 1 (20.0) | 1 (5.9) | 2 (9.1) | 0.937 |
| Rehabilitation | yes | 1 (20.0) | 6 (35.3) | 7 (31.8) | 0.921 |
|  |  |  |  |  |  |

***Abbreviations: CPG: clinical practice guidelines; MDT: multidisciplinary team***

# **Table S9.** Summary of the AGREE-II instruments by items across the different reviewers and average scores for each reviewer

| **Study name** | **AGREE-II instruments** | | | | | | | | | | | | | | | | | | | | | | | |  |
| --- | --- | --- | --- | --- | --- | --- | --- | --- | --- | --- | --- | --- | --- | --- | --- | --- | --- | --- | --- | --- | --- | --- | --- | --- | --- |
|  | **1** | **2** | **3** | **4** | **5** | **6** | **7** | **8** | **9** | **10** | **11** | **12** | **13** | **14** | **15** | **16** | **17** | **18** | **19** | **20** | **21** | **22** | **23** | **Average** | |
| **REVIEWER 1** |  |  |  |  |  |  |  |  |  |  |  |  |  |  |  |  |  |  |  |  |  |  |  |  | |
| Nathens 2004 | 6 | 6 | 7 | 5 | 5 | 5 | 6 | 2 | 5 | 3 | 3 | 3 | 3 | 4 | 1 | 2 | 1 | 4 | 4 | 3 | 4 | 3 | 6 | 57% | |
| Johnson 2005 | 6 | 6 | 5 | 5 | 4 | 6 | 5 | 5 | 5 | 5 | 6 | 6 | 5 | 4 | 6 | 6 | 6 | 5 | 6 | 5 | 5 | 6 | 5 | 72% | |
| Otsuki 2006 | 6 | 5 | 5 | 7 | 3 | 6 | 5 | 3 | 3 | 5 | 6 | 4 | 3 | 2 | 6 | 5 | 6 | 4 | 3 | 3 | 2 | 5 | 3 | 56% | |
| Bailie 2007 | 6 | 6 | 6 | 5 | 3 | 6 | 5 | 4 | 4 | 4 | 6 | 5 | 4 | 3 | 6 | 6 | 7 | 4 | 5 | 4 | 3 | 6 | 4 | 64% | |
| Caroll 2007 | 6 | 5 | 6 | 5 | 3 | 6 | 7 | 5 | 5 | 5 | 6 | 6 | 4 | 3 | 6 | 6 | 7 | 4 | 5 | 4 | 3 | 6 | 4 | 68% | |
| Pezzili 2008 | 6 | 6 | 5 | 5 | 3 | 6 | 6 | 5 | 4 | 5 | 6 | 6 | 4 | 3 | 6 | 5 | 7 | 4 | 5 | 4 | 3 | 6 | 5 | 67% | |
| Pezzilli 2010 | 6 | 5 | 7 | 6 | 6 | 5 | 6 | 2 | 5 | 5 | 5 | 5 | 5 | 4 | 6 | 5 | 1 | 7 | 6 | 5 | 5 | 3 | 6 | 76% | |
| Besselink 2013 | 6 | 7 | 5 | 6 | 6 | 5 | 6 | 2 | 6 | 6 | 6 | 5 | 7 | 5 | 5 | 6 | 1 | 6 | 6 | 4 | 4 | 7 | 6 | 83% | |
| Poma 2013 | 7 | 7 | 6 | 6 | 5 | 5 | 5 | 2 | 5 | 5 | 4 | 4 | 4 | 5 | 5 | 5 | 1 | 4 | 4 | 5 | 4 | 3 | 6 | 72% | |
| Tenner 2013 | 7 | 7 | 7 | 6 | 4 | 7 | 7 | 6 | 6 | 7 | 7 | 7 | 6 | 4 | 7 | 7 | 7 | 5 | 6 | 5 | 4 | 6 | 6 | 86% | |
| Pezzilli 2015 | 7 | 7 | 3 | 6 | 6 | 5 | 6 | 2 | 6 | 6 | 5 | 5 | 5 | 5 | 6 | 6 | 2 | 5 | 6 | 4 | 5 | 2 | 6 | 78% | |
| Greenberg 2016 | 7 | 6 | 4 | 6 | 6 | 5 | 7 | 2 | 5 | 6 | 5 | 5 | 4 | 5 | 4 | 6 | 1 | 5 | 4 | 5 | 4 | 2 | 6 | 72% | |
| Rosolowski 2016 | 6 | 6 | 7 | 4 | 5 | 5 | 4 | 2 | 4 | 2 | 4 | 5 | 5 | 4 | 5 | 4 | 1 | 5 | 5 | 4 | 5 | 2 | 2 | 62% | |
| Crockett 2018 | 6 | 5 | 5 | 6 | 7 | 7 | 5 | 2 | 5 | 3 | 3 | 4 | 4 | 5 | 5 | 4 | 1 | 6 | 6 | 4 | 3 | 4 | 4 | 70% | |
| Leppaniemi 2019 | 5 | 5 | 4 | 6 | 7 | 5 | 5 | 2 | 5 | 3 | 4 | 5 | 3 | 6 | 6 | 5 | 2 | 4 | 3 | 6 | 4 | 6 | 6 | 72% | |
| Vivian 2019 | 6 | 4 | 5 | 7 | 6 | 6 | 7 | 2 | 6 | 7 | 7 | 7 | 7 | 6 | 7 | 7 | 1 | 6 | 7 | 6 | 7 | 6 | 5 | 94% | |
| Arvanitakis 2020 | 5 | 5 | 5 | 7 | 7 | 6 | 7 | 2 | 6 | 7 | 7 | 7 | 7 | 6 | 7 | 6 | 1 | 7 | 7 | 6 | 5 | 5 | 5 | 92% | |
| Liao 2020 | 6 | 4 | 4 | 5 | 6 | 4 | 5 | 2 | 4 | 5 | 5 | 5 | 3 | 6 | 5 | 5 | 1 | 7 | 7 | 3 | 4 | 7 | 7 | 71% | |
| Li 2021 | 7 | 6 | 7 | 6 | 5 | 6 | 6 | 5 | 5 | 6 | 5 | 4 | 6 | 6 | 6 | 7 | 5 | 5 | 4 | 5 | 4 | 4 | 4 | 73% | |
| Takada 2022 | 6 | 5 | 4 | 7 | 6 | 6 | 7 | 2 | 7 | 6 | 6 | 5 | 6 | 6 | 7 | 6 | 1 | 7 | 5 | 5 | 5 | 5 | 7 | 88% | |
| Lee 2023 | 7 | 7 | 7 | 6 | 5 | 7 | 7 | 6 | 5 | 6 | 6 | 5 | 7 | 7 | 6 | 7 | 5 | 5 | 4 | 6 | 6 | 6 | 6 | 84% | |
| **REVIEWER 2** |  |  |  |  |  |  |  |  |  |  |  |  |  |  |  |  |  |  |  |  |  |  |  |  | |
| Nathens 2004 | 5 | 5 | 5 | 6 | 2 | 5 | 3 | 3 | 3 | 3 | 4 | 1 | 2 | 1 | 4 | 4 | 4 | 3 | 3 | 3 | 4 | 3 | 6 | 43% | |
| Johnson 2005 | 6 | 6 | 5 | 7 | 2 | 6 | 3 | 4 | 5 | 6 | 6 | 6 | 6 | 4 | 6 | 6 | 6 | 4 | 5 | 4 | 6 | 7 | 7 | 72% | |
| Otsuki 2006 | 6 | 5 | 5 | 7 | 2 | 7 | 5 | 4 | 4 | 4 | 5 | 3 | 5 | 1 | 4 | 5 | 5 | 4 | 4 | 3 | 2 | 4 | 4 | 54% | |
| Bailie 2007 | 6 | 6 | 6 | 5 | 2 | 7 | 5 | 4 | 5 | 5 | 4 | 4 | 5 | 1 | 5 | 6 | 7 | 5 | 4 | 4 | 2 | 4 | 4 | 60% | |
| Caroll 2007 | 3 | 6 | 5 | 4 | 2 | 4 | 4 | 4 | 6 | 3 | 6 | 5 | 5 | 2 | 4 | 5 | 4 | 5 | 6 | 5 | 2 | 4 | 4 | 54% | |
| Pezzili 2008 | 6 | 6 | 6 | 4 | 2 | 5 | 6 | 5 | 5 | 5 | 5 | 6 | 5 | 1 | 4 | 5 | 4 | 4 | 3 | 4 | 1 | 4 | 4 | 56% | |
| Pezzilli 2010 | 6 | 6 | 5 | 6 | 2 | 5 | 5 | 5 | 5 | 5 | 4 | 6 | 5 | 1 | 7 | 4 | 6 | 4 | 5 | 4 | 5 | 3 | 6 | 63% | |
| Besselink 2013 | 6 | 6 | 5 | 6 | 2 | 6 | 6 | 6 | 5 | 7 | 5 | 5 | 6 | 1 | 6 | 5 | 6 | 5 | 4 | 5 | 4 | 7 | 6 | 70% | |
| Poma 2013 | 6 | 5 | 5 | 5 | 2 | 5 | 5 | 4 | 4 | 4 | 5 | 5 | 5 | 1 | 4 | 5 | 4 | 5 | 5 | 5 | 4 | 3 | 6 | 57% | |
| Tenner 2013 | 7 | 7 | 5 | 5 | 2 | 6 | 7 | 6 | 5 | 7 | 7 | 7 | 6 | 1 | 7 | 7 | 7 | 4 | 5 | 4 | 2 | 7 | 7 | 76% | |
| Pezzilli 2015 | 6 | 6 | 5 | 6 | 2 | 6 | 6 | 5 | 5 | 5 | 5 | 6 | 6 | 2 | 5 | 5 | 6 | 5 | 4 | 4 | 5 | 2 | 6 | 65% | |
| Greenberg 2016 | 6 | 6 | 5 | 7 | 2 | 5 | 6 | 5 | 5 | 4 | 5 | 4 | 6 | 1 | 5 | 4 | 4 | 4 | 5 | 5 | 4 | 2 | 6 | 60% | |
| Rosolowski 2016 | 4 | 5 | 5 | 4 | 2 | 4 | 2 | 4 | 5 | 5 | 4 | 5 | 4 | 1 | 5 | 4 | 5 | 4 | 4 | 5 | 5 | 2 | 2 | 49% | |
| Crockett 2018 | 6 | 7 | 7 | 5 | 2 | 5 | 3 | 3 | 4 | 4 | 5 | 5 | 4 | 1 | 6 | 5 | 6 | 5 | 4 | 5 | 3 | 4 | 4 | 58% | |
| Leppaniemi 2019 | 6 | 7 | 5 | 5 | 2 | 5 | 3 | 4 | 5 | 3 | 6 | 6 | 5 | 2 | 4 | 6 | 3 | 5 | 6 | 5 | 4 | 6 | 6 | 62% | |
| Vivian 2019 | 7 | 6 | 6 | 7 | 2 | 6 | 7 | 7 | 7 | 7 | 6 | 7 | 7 | 1 | 6 | 6 | 7 | 7 | 6 | 5 | 7 | 6 | 5 | 83% | |
| Arvanitakis 2020 | 7 | 7 | 6 | 7 | 2 | 6 | 7 | 7 | 7 | 7 | 6 | 7 | 6 | 1 | 7 | 6 | 7 | 6 | 6 | 5 | 5 | 5 | 5 | 81% | |
| Liao 2020 | 5 | 6 | 4 | 5 | 2 | 4 | 5 | 5 | 5 | 3 | 6 | 5 | 5 | 1 | 7 | 5 | 7 | 3 | 3 | 3 | 4 | 7 | 7 | 61% | |
| Li 2021 | 7 | 6 | 7 | 6 | 2 | 6 | 5 | 5 | 5 | 5 | 4 | 6 | 6 | 1 | 7 | 5 | 7 | 4 | 4 | 5 | 4 | 6 | 7 | 70% | |
| Takada 2022 | 7 | 6 | 6 | 7 | 2 | 7 | 6 | 6 | 5 | 6 | 6 | 7 | 6 | 1 | 7 | 5 | 5 | 6 | 5 | 6 | 5 | 5 | 7 | 77% | |
| Lee 2023 | 7 | 6 | 7 | 7 | 2 | 7 | 5 | 6 | 5 | 6 | 5 | 5 | 5 | 1 | 6 | 5 | 7 | 5 | 5 | 4 | 4 | 4 | 7 | 71% | |
| **REVIEWER 3** |  |  |  |  |  |  |  |  |  |  |  |  |  |  |  |  |  |  |  |  |  |  |  |  | |
| Nathens 2004 | 5 | 4 | 6 | 7 | 1 | 4 | 7 | 7 | 7 | 7 | 4 | 1 | 7 | 1 | 4 | 4 | 5 | 2 | 2 | 1 | 1 | 1 | 1 | 48% | |
| Johnson 2005 | 7 | 7 | 7 | 7 | 5 | 6 | 1 | 4 | 1 | 5 | 6 | 6 | 7 | 7 | 6 | 7 | 6 | 6 | 4 | 1 | 7 | 7 | 7 | 75% | |
| Otsuki 2006 | 7 | 7 | 7 | 7 | 1 | 7 | 5 | 1 | 5 | 2 | 6 | 6 | 7 | 1 | 6 | 4 | 3 | 5 | 5 | 1 | 1 | 4 | 1 | 55% | |
| Bailie 2007 | 7 | 7 | 7 | 7 | 2 | 7 | 5 | 1 | 4 | 3 | 4 | 1 | 7 | 1 | 6 | 6 | 7 | 3 | 1 | 1 | 1 | 3 | 1 | 50% | |
| Caroll 2007 | 1 | 1 | 1 | 7 | 1 | 3 | 1 | 2 | 4 | 2 | 5 | 6 | 7 | 1 | 4 | 5 | 3 | 3 | 6 | 2 | 1 | 7 | 1 | 37% | |
| Pezzili 2008 | 2 | 2 | 2 | 7 | 1 | 2 | 1 | 1 | 3 | 6 | 5 | 7 | 7 | 1 | 5 | 6 | 5 | 3 | 3 | 1 | 1 | 7 | 7 | 45% | |
| Pezzilli 2010 | 6 | 6 | 6 | 7 | 1 | 5 | 7 | 6 | 5 | 6 | 3 | 6 | 7 | 1 | 7 | 5 | 6 | 2 | 2 | 1 | 6 | 4 | 7 | 64% | |
| Besselink 2013 | 7 | 7 | 6 | 7 | 1 | 7 | 7 | 7 | 6 | 7 | 5 | 7 | 7 | 1 | 7 | 6 | 7 | 5 | 5 | 6 | 6 | 7 | 7 | 83% | |
| Poma 2013 | 6 | 7 | 5 | 7 | 1 | 6 | 7 | 6 | 6 | 7 | 6 | 6 | 7 | 1 | 7 | 4 | 5 | 4 | 4 | 5 | 1 | 7 | 2 | 68% | |
| Tenner 2013 | 7 | 7 | 7 | 7 | 1 | 1 | 6 | 6 | 6 | 7 | 7 | 7 | 7 | 1 | 7 | 7 | 7 | 4 | 3 | 1 | 1 | 7 | 7 | 71% | |
| Pezzilli 2015 | 7 | 7 | 2 | 7 | 1 | 3 | 7 | 5 | 6 | 6 | 5 | 7 | 7 | 5 | 7 | 7 | 6 | 2 | 2 | 1 | 5 | 1 | 7 | 65% | |
| Greenberg 2016 | 7 | 7 | 4 | 7 | 1 | 5 | 6 | 6 | 6 | 5 | 4 | 6 | 7 | 1 | 6 | 6 | 6 | 4 | 5 | 6 | 5 | 2 | 7 | 70% | |
| Rosolowski 2016 | 1 | 1 | 3 | 7 | 1 | 4 | 1 | 1 | 4 | 5 | 4 | 5 | 7 | 1 | 6 | 5 | 7 | 4 | 3 | 1 | 1 | 1 | 1 | 37% | |
| Crockett 2018 | 7 | 7 | 7 | 7 | 1 | 6 | 7 | 7 | 7 | 7 | 6 | 7 | 7 | 1 | 6 | 6 | 7 | 6 | 5 | 3 | 1 | 7 | 7 | 79% | |
| Leppaniemi 2019 | 7 | 7 | 5 | 7 | 1 | 5 | 1 | 1 | 4 | 5 | 6 | 6 | 7 | 1 | 4 | 5 | 2 | 4 | 5 | 6 | 1 | 7 | 7 | 59% | |
| Vivian 2019 | 7 | 7 | 6 | 7 | 1 | 7 | 7 | 7 | 7 | 7 | 6 | 7 | 7 | 1 | 6 | 6 | 7 | 7 | 5 | 6 | 4 | 7 | 4 | 82% | |
| Arvanitakis 2020 | 6 | 7 | 6 | 7 | 1 | 6 | 7 | 7 | 7 | 7 | 6 | 7 | 7 | 1 | 7 | 7 | 7 | 6 | 6 | 7 | 1 | 4 | 4 | 78% | |
| Liao 2020 | 4 | 5 | 5 | 7 | 1 | 5 | 4 | 2 | 5 | 4 | 6 | 4 | 7 | 1 | 7 | 5 | 7 | 2 | 3 | 1 | 1 | 7 | 7 | 56% | |
| Li 2021 | 7 | 6 | 6 | 7 | 1 | 6 | 1 | 1 | 5 | 5 | 3 | 6 | 7 | 1 | 7 | 6 | 7 | 4 | 4 | 4 | 1 | 7 | 7 | 62% | |
| Takada 2022 | 7 | 6 | 7 | 7 | 1 | 7 | 7 | 7 | 6 | 7 | 6 | 7 | 7 | 1 | 7 | 6 | 7 | 6 | 7 | 6 | 1 | 2 | 7 | 79% | |
| Lee 2023 | 7 | 7 | 7 | 7 | 1 | 7 | 7 | 5 | 6 | 7 | 5 | 7 | 7 | 1 | 6 | 6 | 7 | 3 | 4 | 1 | 1 | 7 | 7 | 72% | |
| **REVIEWER 4** |  |  |  |  |  |  |  |  |  |  |  |  |  |  |  |  |  |  |  |  |  |  |  |  | |
| Nathens 2004 | 5 | 4 | 6 | 7 | 1 | 4 | 7 | 7 | 7 | 7 | 4 | 1 | 7 | 1 | 4 | 4 | 5 | 2 | 2 | 1 | 1 | 1 | 1 | 48% | |
| Johnson 2005 | 7 | 7 | 7 | 7 | 5 | 6 | 1 | 4 | 1 | 5 | 6 | 6 | 7 | 7 | 6 | 7 | 6 | 6 | 4 | 1 | 7 | 7 | 7 | 75% | |
| Otsuki 2006 | 7 | 7 | 7 | 7 | 1 | 7 | 5 | 1 | 5 | 2 | 6 | 6 | 7 | 1 | 6 | 4 | 3 | 5 | 5 | 1 | 1 | 4 | 1 | 55% | |
| Bailie 2007 | 7 | 7 | 7 | 7 | 2 | 7 | 5 | 1 | 4 | 3 | 4 | 1 | 7 | 1 | 6 | 6 | 7 | 3 | 1 | 1 | 1 | 3 | 1 | 50% | |
| Caroll 2007 | 1 | 1 | 1 | 7 | 1 | 3 | 1 | 2 | 4 | 2 | 5 | 6 | 7 | 1 | 4 | 5 | 3 | 3 | 6 | 2 | 1 | 7 | 1 | 37% | |
| Pezzili 2008 | 2 | 2 | 2 | 7 | 1 | 2 | 1 | 1 | 3 | 6 | 5 | 7 | 7 | 1 | 5 | 6 | 5 | 3 | 3 | 1 | 1 | 7 | 7 | 45% | |
| Pezzilli 2010 | 6 | 6 | 6 | 7 | 1 | 5 | 7 | 6 | 5 | 6 | 3 | 6 | 7 | 1 | 7 | 5 | 6 | 2 | 2 | 1 | 6 | 4 | 7 | 64% | |
| Besselink 2013 | 7 | 7 | 6 | 7 | 1 | 7 | 7 | 7 | 6 | 7 | 5 | 7 | 7 | 1 | 7 | 6 | 7 | 5 | 5 | 6 | 6 | 7 | 7 | 83% | |
| Poma 2013 | 6 | 7 | 5 | 7 | 1 | 6 | 7 | 6 | 6 | 7 | 6 | 6 | 7 | 1 | 7 | 4 | 5 | 4 | 4 | 5 | 1 | 7 | 2 | 68% | |
| Tenner 2013 | 7 | 7 | 7 | 7 | 1 | 1 | 6 | 6 | 6 | 7 | 7 | 7 | 7 | 1 | 7 | 7 | 7 | 4 | 3 | 1 | 1 | 7 | 7 | 71% | |
| Pezzilli 2015 | 7 | 7 | 2 | 7 | 1 | 3 | 7 | 5 | 6 | 6 | 5 | 7 | 7 | 5 | 7 | 7 | 6 | 2 | 2 | 1 | 5 | 1 | 7 | 65% | |
| Greenberg 2016 | 7 | 7 | 4 | 7 | 1 | 5 | 6 | 6 | 6 | 5 | 4 | 6 | 7 | 1 | 6 | 6 | 6 | 4 | 5 | 6 | 5 | 2 | 7 | 70% | |
| Rosolowski 2016 | 1 | 1 | 3 | 7 | 1 | 4 | 1 | 1 | 4 | 5 | 4 | 5 | 7 | 1 | 6 | 5 | 7 | 4 | 3 | 1 | 1 | 1 | 1 | 37% | |
| Crockett 2018 | 7 | 7 | 7 | 7 | 1 | 6 | 7 | 7 | 7 | 7 | 6 | 7 | 7 | 1 | 6 | 6 | 7 | 6 | 5 | 3 | 1 | 7 | 7 | 79% | |
| Leppaniemi 2019 | 7 | 7 | 5 | 7 | 1 | 5 | 1 | 1 | 4 | 5 | 6 | 6 | 7 | 1 | 4 | 5 | 2 | 4 | 5 | 6 | 1 | 7 | 7 | 59% | |
| Vivian 2019 | 7 | 7 | 6 | 7 | 1 | 7 | 7 | 7 | 7 | 7 | 6 | 7 | 7 | 1 | 6 | 6 | 7 | 7 | 5 | 6 | 4 | 7 | 4 | 82% | |
| Arvanitakis 2020 | 6 | 7 | 6 | 7 | 1 | 6 | 7 | 7 | 7 | 7 | 6 | 7 | 7 | 1 | 7 | 7 | 7 | 6 | 6 | 7 | 1 | 4 | 4 | 78% | |
| Liao 2020 | 4 | 5 | 5 | 7 | 1 | 5 | 4 | 2 | 5 | 4 | 6 | 4 | 7 | 1 | 7 | 5 | 7 | 2 | 3 | 1 | 1 | 7 | 7 | 56% | |
| Li 2021 | 7 | 6 | 6 | 7 | 1 | 6 | 1 | 1 | 5 | 5 | 3 | 6 | 7 | 1 | 7 | 6 | 7 | 4 | 4 | 4 | 1 | 7 | 7 | 62% | |
| Takada 2022 | 7 | 6 | 7 | 7 | 1 | 7 | 7 | 7 | 6 | 7 | 6 | 7 | 7 | 1 | 7 | 6 | 7 | 6 | 7 | 6 | 1 | 2 | 7 | 79% | |
| Lee 2023 | 7 | 7 | 7 | 7 | 1 | 7 | 7 | 5 | 6 | 7 | 5 | 7 | 7 | 1 | 6 | 6 | 7 | 3 | 4 | 1 | 1 | 7 | 7 | 72% | |
